# Bimatoprost-Loaded Ocular Inserts as Sustained Release Drug Delivery Systems for Glaucoma Treatment: *In Vitro* and *In Vivo* Evaluation

**DOI:** 10.1371/journal.pone.0095461

**Published:** 2014-04-30

**Authors:** Juçara Ribeiro Franca, Giselle Foureaux, Leonardo Lima Fuscaldi, Tatiana Gomes Ribeiro, Lívia Bomfim Rodrigues, Renata Bravo, Rachel Oliveira Castilho, Maria Irene Yoshida, Valbert Nascimento Cardoso, Simone Odília Fernandes, Sebastião Cronemberger, Anderson José Ferreira, André Augusto Gomes Faraco

**Affiliations:** 1 Department of Pharmaceutical Products, Federal University of Minas Gerais, Belo Horizonte, Minas Gerais, Brazil; 2 Department of Morphology, Federal University of Minas Gerais, Belo Horizonte, Minas Gerais, Brazil; 3 Department of Clinical and Toxicological Analysis, Federal University of Minas Gerais, Belo Horizonte, Minas Gerais, Brazil; 4 Department of Chemistry, Federal University of Minas Gerais, Belo Horizonte, Minas Gerais, Brazil; 5 Department of Ophthalmology and Otolaryngology, Federal University of Minas Gerais, Belo Horizonte, Minas Gerais, Brazil; Université de Lorraine, France

## Abstract

The purpose of the present study was to develop and assess a novel sustained-release drug delivery system of Bimatoprost (BIM). Chitosan polymeric inserts were prepared using the solvent casting method and characterized by swelling studies, infrared spectroscopy, differential scanning calorimetry, drug content, scanning electron microscopy and *in vitro* drug release. Biodistribution of ^99m^Tc-BIM eye drops and ^99m^Tc-BIM-loaded inserts, after ocular administration in Wistar rats, was accessed by *ex vivo* radiation counting. The inserts were evaluated for their therapeutic efficacy in glaucomatous Wistar rats. Glaucoma was induced by weekly intracameral injection of hyaluronic acid. BIM-loaded inserts (equivalent to 9.0 µg BIM) were administered once into conjunctival sac, after ocular hypertension confirmation. BIM eye drop was topically instilled in a second group of glaucomatous rats for 15 days days, while placebo inserts were administered once in a third group. An untreated glaucomatous group was used as control. Intraocular pressure (IOP) was monitored for four consecutive weeks after treatment began. At the end of the experiment, retinal ganglion cells and optic nerve head cupping were evaluated in the histological eye sections. Characterization results revealed that the drug physically interacted, but did not chemically react with the polymeric matrix. Inserts sustainedly released BIM *in vitro* during 8 hours. Biodistribution studies showed that the amount of ^99m^Tc-BIM that remained in the eye was significantly lower after eye drop instillation than after chitosan insert implantation. BIM-loaded inserts lowered IOP for 4 weeks, after one application, while IOP values remained significantly high for the placebo and untreated groups. Eye drops were only effective during the daily treatment period. IOP results were reflected in RGC counting and optic nerve head cupping damage. BIM-loaded inserts provided sustained release of BIM and seem to be a promising system for glaucoma management.

## Introduction

Glaucoma is an ocular disorder with multi-factorial etiology, characterized by a slow progressive degeneration of retinal ganglion cells (RGC) and optic nerve axons [1], [2]. The disease is the second leading cause of blindness and, worldwide, it is estimated that about 66.8 million people have contracted some form of visual impairment from glaucoma, with 6.7 million suffering from blindness [3]–[5]. The most important risk factor for glaucoma is intraocular pressure (IOP) elevation; progressive visual loss is associated with increased IOP, which damages the optic nerve [6]–[8]. Primary open-angle glaucoma (POAG) is the most common type of glaucoma [9]. In POAG it is not possible to establish a clinical cause to neurodegeneration or to IOP elevation [10], [11]. POAG therapy usually begins with medications [3].

Prostaglandin analogs have become the first therapeutic class of choice for medical treatment of POAG because of their improved efficacy and tolerability [12], [13]. Prostaglandin analogs are able to control IOP, primarily by increasing uveoscleral outflow via remodeling of the ciliary body [14]. Bimatoprost (BIM) is a prostaglandin analog chemically related to prostamide F_2α_ [15]. The mechanism of IOP lowering induced by BIM is not completely understood. Some authors suggest that BIM is an agonist to “prostamide” receptor [16]–[18], while another study in human eye tissues has shown that BIM is rapidly hydrolyzed in cornea, iris, sclera, and ciliary muscle to its corresponding 17-phenyl-prostaglandin F_2α_ metabolite (free acid), known to be active at the prostaglandin F receptor [19]. Another recent work also suggested that both BIM and its metabolite promote protective effects on the RCG against oxidative stress [14].

Eye drops are still the mainstay eye disease management, accounting for approximately 90% of all ophthalmic treatments. Nevertheless, only 1% to 7% of the administered drugs actually reach the aqueous humor [20]–[22]. The inefficiency of this route stems mainly from the precorneal tear clearance mechanism, the highly selective corneal epithelial barrier, and patient compliance, a factor that is quite unpredictable and difficult to control [22]. Clinically, in glaucomatous patients, variable bioavailability of the active ingredients results in diurnal IOP fluctuation [23]. Moreover, patients tend to present inadequate adherence to their daily therapeutic regimen [23]. Altogether, these factors may result in therapeutic inefficacy and progression of the glaucoma damage. Therefore, the development of new vehicles and drug formulations that require less patient effort and enhance the drug bioavailability represent an important aspect in controlling the evolution this disease [22].

Sustained-release drug delivery systems have been developed to overcome eye drops limitations [24]. These systems can achieve prolonged therapeutic drug concentrations in ocular target tissues, while limiting systemic exposure and side effects and improving patient adherence to therapy [25]. Ocular inserts are solid or semi-solid devices meant to be placed in the conjunctival sac (between the lower eyelid and the eye itself) to deliver drugs on the ocular surface [26]. These devices are designed to release the drug at a constant rate for a prolonged time while minimizing systemic absorption through the nasal mucosa and improving patient compliance due to a reduced frequency of administration. Inserts are often matrix based and made of degradable polymers, such as Chitosan (CS) [22], [27].

CS is a natural carbohydrate polymer, has number of applications in the field of ophthalmics and has attracted a great deal of attention from the scientific community and environmentalists due to its unique features [28]. CS is a biodegradable, nontoxic, and biocompatible polymer [29], which can enhance the intraocular bioavailability of both hydrophilic and lipophilic drugs [29], [30]. Moreover, CS is a promising mucoadhesive material in physiological pH [31]–[34]. Due to interactions with the mucus layer or the eye tissues, an increase in the precorneal residence time of the preparation can be observed, leading to an increase in the bioavailability of the drug instilled [21]. Thus, mucoadhesive ocular inserts (such as CS-based inserts) could increase the precorneal drug retention (to delay washout), which may result in an enhanced formulation bioavailability.

In this regard, to improve patient compliance by lowering the frequency of administration and to enhance therapeutic effectiveness of glaucoma medical treatment, this research aimed at formulating, physicochemically and *in vivo* evaluating CS-based inserts for a sustained release of BIM, which were able to lower IOP over four weeks after one application, without causing any damage to the animals.

## Materials and Methods

### Materials

Medium molecular weight CS and Bimatoprost were supplied by Sigma-Aldrich. All other reagents were of analytical grade.

### Preparation of BIM-loaded inserts

Inserts were prepared as monolayer films by employing the solvent/casting technique, according to Rodrigues *et al.* [35]. First, 75 µL of acetic acid was added to 5 mL of an aqueous solution containing 1.5 mg of BIM homogeneously dissolved. Next, 250 mg of CS was added in this solution. This viscous dispersion was magnetically stirred overnight to ensure homogeneity of both drug and polymer. Then, it was cast, at room temperature, in circular silicone-molded trays (SMT) containing individual 5 mm×2 mm wells [36] to produce BIM-loaded insert (BI). After casting, inserts were carefully removed from the SMT and stored in receptacles, protected from light and air humidity. Placebo inserts (PI) were produced in a similar manner, by adding 250 mg of CS to 5 mL of a solution containing 75 µL of acetic acid in water.

### Characterization studies

#### Swelling studies

Inserts swelling studies were carried out in a phosphate buffer solution (pH 7.4) (PBS). Each insert was weighed and placed in PBS for predetermined periods of time (5, 10, 20, 40, 60, and 90 min) as described by Eroglu *et al.* [37]. After immersion, the inserts were removed from the medium, the excess surface water was removed by using filter paper, and the pieces were weighed. The degree of swelling was calculated by using the equation: *Swellingindex*  =  [(*W_t_-W_0_*)/*W_0_*]. [38].

The weight of the swollen insert after predetermined periods of time (*t*) is represented by *W_t_*. The original weight of the insert at zero time is represented by *W_0_*. This experiment was performed in triplicate.

#### ATR-FTIR analysis

Attenuated Total Reflectance Fourier Transformed Infrared spectroscopy (ATR-FTIR) spectra of all the inserts and of the original polymer powders were recorded on a PerkinElmer FTIR Spectrometer, Model Spectrum One (USA).

#### DSC analysis

Thermal properties of the inserts were also evaluated. Differential Scanning Calorimetry (DSC) measurements were carried out in a Shimadzu DSC50. Samples (inserts, powder CS, and powder HEC) were packed in an aluminum crucible and heated at a rate of 10°C/min. Nitrogen, at the rate of 20 mL/min was used as a purge gas during the role analysis. The specimens were heated from −50°C to 200°C (RUN 1). The specimens were then cooled to −50°C at the same rate of 10°C/min, at which point they were reheated to 400°C at a rate of 10°C/min (RUN 2).

#### SEM analysis

The inserts morphology was studied using a JEOL scanning electron microscope (SEM), model JSM-6360LV, operating at 15 kV. The samples were prepared by freezing the inserts in liquid nitrogen. Then, the inserts were fractured. Next, the surface and sides of the inserts were analyzed. The devices were analyzed at suitable acceleration voltages using varying magnification for each sample. Representative micrographs were also taken.

#### Determination of BIM

The high performance liquid chromatography (HPLC) method was chosen to quantify the amount of BIM loaded in the inserts. A Waters HPLC with Pump Control Module (PCM II), a Binary Pump System Waters 515 CLAE Pump, a Waters 717 Plus Autosampler, and a Waters 486 Turnable Absorbance Detector were used. Merck Column Lichro CART 100 (ODS) of 250×4.6 mm, with a particle size of 5 µm, was used for the stationary phase at room temperature. The mobile phase, at a flow rate of 1.0 mL/min, was comprised of methanol/acetonitrile/phosphoric acid at 0.1 vol% (30∶30∶40), and detection was performed at 210 nm. The solvents were HPLC grade (Tédia Brazil). Samples were dissolved in PBS. After filtration on a 0.45 µm membrane made of regenerated cellulose (Sartorius, Sweden), 20 µL of the samples were automatically injected into the apparatus. The method was validated in accordance with ICH guidelines. BIM, at a concentration of 3.0–15 µg/mL in PBS, was used as a standard solution (phosphate buffer solution, pH 7.4) (y = 26433.5x+880.449, R^2^ = 0.9994; n = 3).

#### Drug Content uniformity

Drug Content uniformity was performed according to Brazilian Pharmacopeia [39]. Each ocular insert was hydrated with 100 µL of acetic acid. After 30 minutes, 900 µL of water was added to dissolve the insert. The solution was filtered and suitably diluted. The BIM content was analyzed by HPLC. This test was performed on 10 ocular inserts.

#### *In vitro* drug release

*In vitro* drug release was evaluated using the Franz cell system. A cellulose acetate membrane with 0.45 µm pores was used to split the insert compartment from the receptor liquid compartment. PBS was used as a receptor liquid, and the glass cells were incubated at 37±0.5°C. At appropriate time intervals, all the receptor liquid was withdrawn from the glass cells, and an equal volume of the same receptor liquid was added to maintain a constant volume. The amount of drug released was evaluated by HPLC. The experiment was performed in quintuplicate.

### In vivo studies

#### Animals

Male Wistar rats weighing 180 to 220 g were obtained from the animal facility of the Faculty of Pharmacy, Federal University of Minas Gerais. The animals were housed in a temperature-controlled room (22–23 °C) with a 12–12 h light-dark cycle. Water and food were available *ad libitum*. The experimental protocols were performed and approved in accordance with institutional guidelines by the Ethics Committee in Animal Experimentation of the Federal University of Minas Gerais, Brazil (CETEA-UFMG), which are in accordance with the National Institutes of Health (NIH) Guidelines for the Care and Use of Laboratory Animals (protocols n° 251/11 and 211/13). In addition, this study conforms to the Association for Research in Vision and Ophthalmology (ARVO) statement for the use of animals in ophthalmic and vision research.

#### Biodistribution studies

Biodistribution studies were based on the administration of free and entrapped BIM, radiolabeled with technetium-99m (^99m^Tc). The method for ^99m^Tc labeling of BIM was developed in our laboratory, based on the radiolabeling protocols developed by Nunan et al. [40] and de Barros et al. [41], with modifications. BIM was dissolved in 1.0 mL of phosphate buffer (pH 7.4) and radiolabeled with ^99m^Tc by direct labeling method using stannous chloride as reducing agent. Purification was processed by the addition of 200 mg of G_60_ silica to the radiolabeled solution. After 15 minutes, suspension was centrifuged at 10,000 rpm during 10 minutes and supernatant was recovered. Radiochemical purity analysis of ^99m^Tc-BIM was performed by instant thin-layer chromatography on silica gel strips (ITLC-SG, Merck), using a two-solvent system: saline and ethyl acetate/acetone (5∶95) to determine the amount of free technetium (^99m^TcO_4_^−^) and hydrolysed technetium (^99m^TcO_2_), respectively. ^99m^Tc-BIM solution was used to prepare BI as described earlier.

^99m^Tc-BIM (free and in the insert) was topically administered in the right eye of Wistar rats (*n* = 5), as decribed above. At 8 and 18 hours post-administration, rats were anesthetized intramuscularly with a solution of ketamine (70 mg/Kg) and xylazine (10 mg/Kg) and, then, euthanized. Organs and tissues of interest (spleen, liver, stomach, small and large intestines, kidneys, blood and eyes) were harvested. Then, each organ and tissue was weighed and its associated radioactivity was determined in an automatic gamma counter (Wizard, Finland). The results were expressed as the percentage of radioactivity per gram of tissue (% cpm/g). Data were statistically analyzed by means of unpaired *t test*, using PRISM 5.0 software.

#### *In vivo* efficacy

Unilateral glaucoma was induced in the right eye by injection of 30 µL of hyaluronic acid (HA) (10 mg/mL) into the anterior chamber through the clear cornea, near the corneoscleral limbus using an hypodermic needle (22 gauge), once a week, for 6 weeks, on the same calendar day and time, according to Moreno et al. [42]. Rats were anesthetized intramuscularly with ketamine (70 mg/kg) and xylazine (10 mg/kg). In addition, two drops of 0.4% benoxinate hydrochloride were instilled directly on the cornea as a local anesthetic. No procedure was performed in the contralateral eye (control groups). Evaluation of IOP and mean arterial pressure (MAP) were carried out one day before the next HA injection.

BI was placed, once, into the conjunctival sac after the establishment of ocular hypertension (immediately after the second induction). A marketed formulation of BIM eye drops were used as positive control. In this case, animals were treated daily during two weeks. The treatment was also started immediately after the second induction. Untreated animals and PI were used as negative controls. PI was also placed once in the conjunctival sac immediately after the second induction. Five animals were used in each group. Both eyes (sick and healthy) of each animal were submitted to the same treatment. *Inserts* were hydrated with saline for 30 seconds before administration.

IOP measurements were performed using an applanation tonometer TonoPen XL (Mentor, Norwell, MA, USA) which was calibrated before use by an experienced person. A topical anesthetic (benoxinate hydrochloride 0.4%) was applied to each eye prior to the measurement of IOP. To obtain the measures, non-sedated animals (n = 5) were carefully contained with a small cloth and three readings of IOP (with standard error less than 10%) were acquired in each eye. The average of these three measures was considered the corresponding value of IOP. IOP measurements were performed at the same time each day or week (between 11:00 AM and 12:00 PM) to correct diurnal variations in IOP. The tonometrist was masked to the treatment and an assistant performed the randomization process.

Mean arterial pressure (MAP) was evaluated by a tail-cuff method, which is a noninvasive computerized system for measuring blood pressure (Kent Scientific Corporation, Torrington, CT, USA). This tail-cuff blood pressure system utilizes volume pressure recording sensor technology to measure the rat tail blood pressure. The animals (n = 5) were acclimated one day before the beginning of the experiments to restraint and to tail-cuff inflation. The restraint platform was maintained at approximately 32–34°C. For each session the rat was placed in an acrylic box restraint and the tail was inserted into a compression cuff that measured the blood pressure 15 times to calculate the average.

Animals were euthanized and both eye were enucleated for histological analysis. After enucleation, two small sagittal sections were made in each side of the eyes. Then, they were immediately immersed in Bouin's fluid for approximately 24 hours. Thereafter, they were dehydrated in increasing concentrations of ethanol (70, 80, 90, 95 and 100%), diaphanized in xylene, and included and embedded in Paraplast. Semi-serial 6 µm-sections (60 µm of interval) were obtained using a microtome (model HM335E, Microm, Minnesota, USA). For histological analysis and RGC counting, histological sections were stained with hematoxylin-eosin (HE).

### Statistical analyses

Data were expressed as mean ± SEM. Comparative results were analyzed using unpaired t test (for Biodistribution) and one-way ANOVA followed by the Tukey post-test (for PIO, MAP and RCG counting). All these tests were performed using the GraphPad Prism 5 software. Results were considered significant at the p<0.05 level.

## Results

### Characterization studies and *in vitro* drug release

Chitosan polymeric inserts were produced as circular flexible films with 5 mm of diameter. Physicochemical interactions between drug and polymeric matrix were evaluated by different techniques. Results of the characterization studies are presented below.

**Figure 1:**
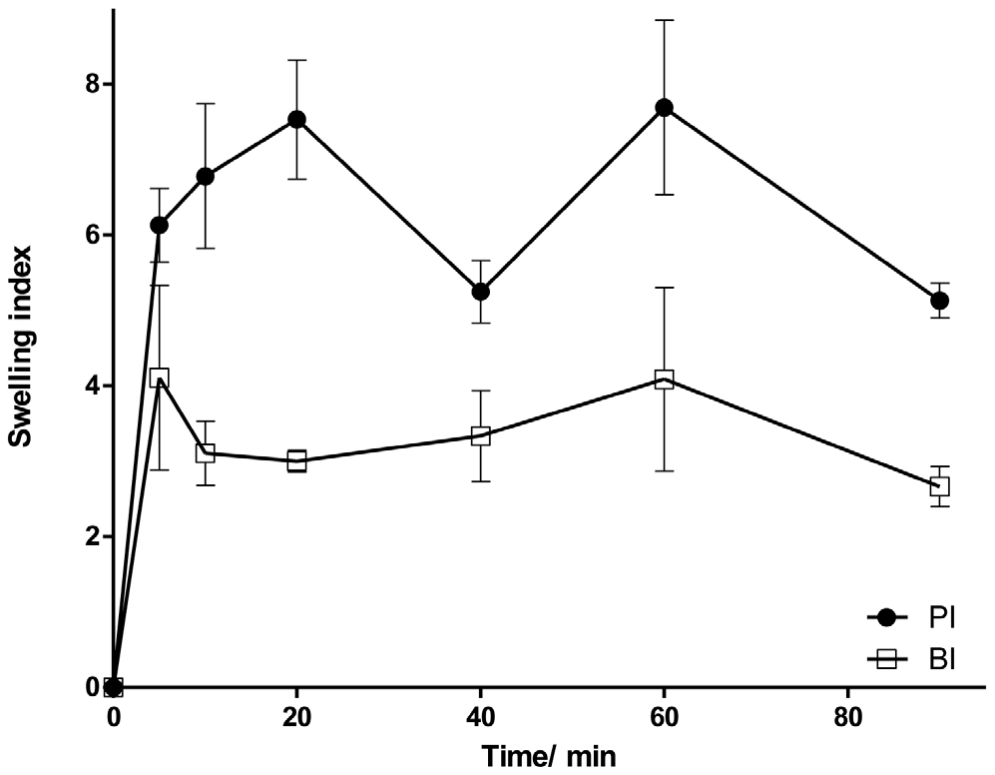
Swelling index of PI and BI in a buffered solution medium (PBS; pH 7.4). Values expressed as mean ± SD.

Swelling indexes of inserts are shown in **Fig. 1**. Inserts hydrated very quickly, reaching more than 80% of total hydration in the first 20 min. The effects of BIM on the swelling behavior are also presented in **Fig. 1**. The water soluble drug (BIM) decreased the water uptake of BI, compared to PI. During the accomplishment of the test, the integrity of the tested films was not lost.

**Figure 2:**
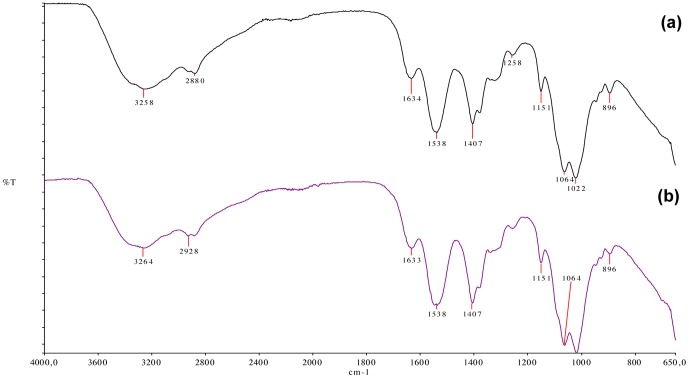
ATR-FTIR spectra of PI (a) and BI (b). BIM shifted first band to a higher frequency (from 3258 to 3264 cm^−1^) and widened (arrow).

**Fig. 2** shows the ATR-FTIR spectra PI and BI. In both spectra, two characteristic absorption bands of CS were detected at 1634 and 1538 cm^−1^ and attributed to amide I (C = O stretching) and to N-H (amine) vibration overlapped to amide II (N-H vibration), respectively. In the FTIR spectrum of PI, the wide and overlapped absorption band at 3258 cm^−1^ was due to the stretching vibration of the O-H and N-H bonds [35], [43], [44]. From the FTIR spectra of BI, it can be seen that the first band shifted to a higher frequency (from 3258 to 3264 cm^−1^) and widened.

**Figure 3:**
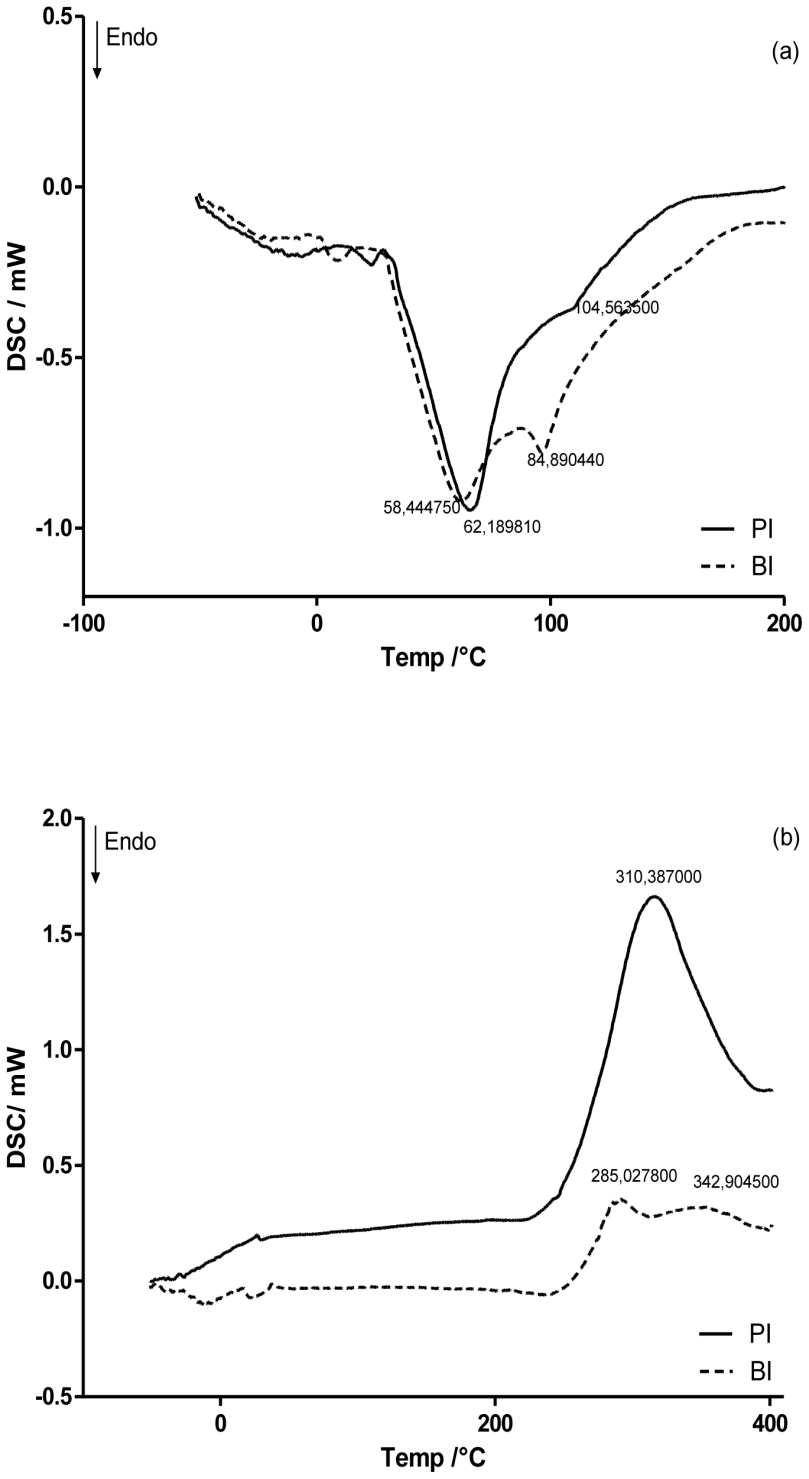
DSC curves of PI and BI. (a) First run; (b) second run.

In **Fig. 3** shows the DSC curves of PI and BI. PI presented a broad endothermic peak at 62.19°C, as well as a broad exothermic peak at 310.39°C on the first and second runs, respectively. Both peaks are irregular and can be attributed to an evaporation of residual water and a degradation of the main chain, respectively [35], [45], [46]. The degradation peak of CS was dismembered and an increase in the degradation temperature from 310.38°C to 342.90°C could be observed when BIM was added to the inserts.

**Figure 4:**
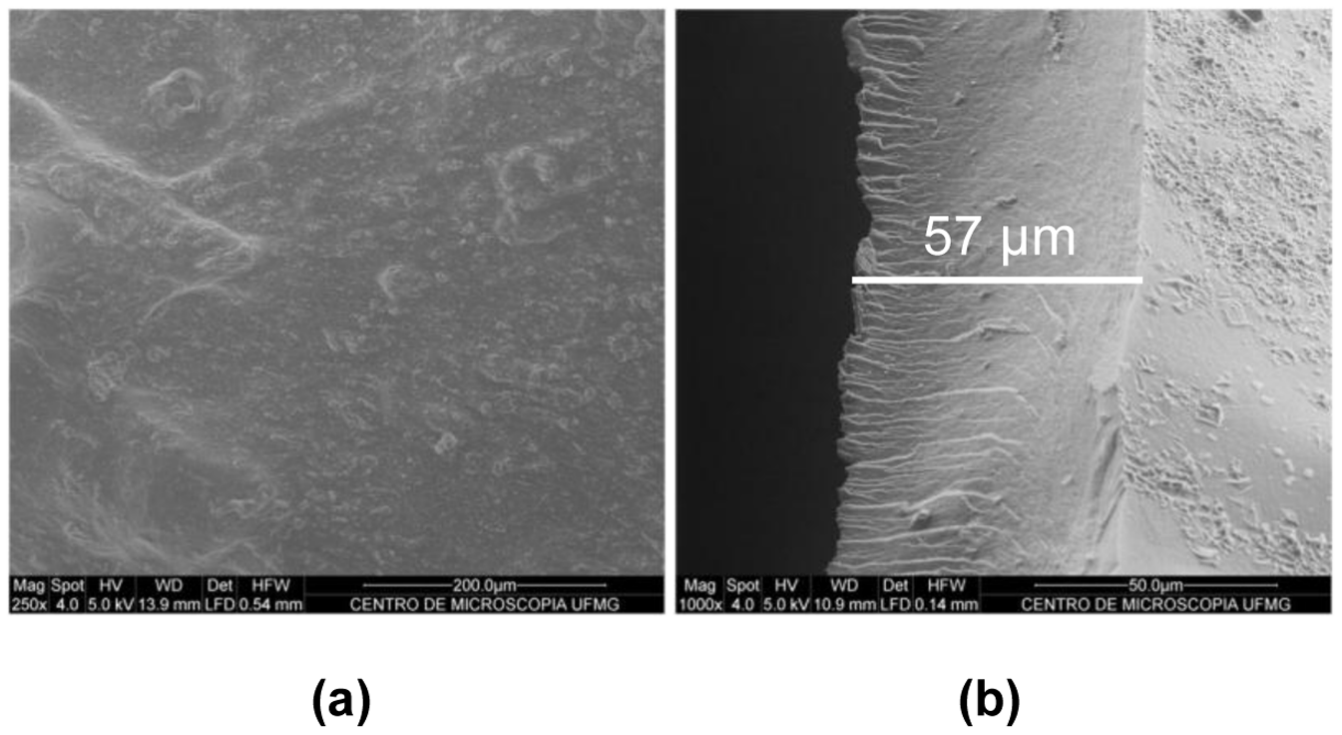
Representative SEM photomicrographs of Bimatoprost-loaded inserts. (a) surface; (b) lateral. Bar indicates the thickness of the insert.

Morphological characterization of inserts was performed by SEM analysis. SEM pictures of PI and BI are shown in **Fig. 4**. From surface images (**Fig. 4** **a**), it is possible to notice that inserts showed an irregular surface. However, it is not possible to identify drug crystals in this surface, suggesting the miscibility of the drug in the polymer matrix. Lateral images (**Fig. 4** **b**) shows that the insert polymeric matrix is homogeneous, compact, without any kind of crystallized or granular particles and have about 50 µm of thickness.

**Figure 5:**
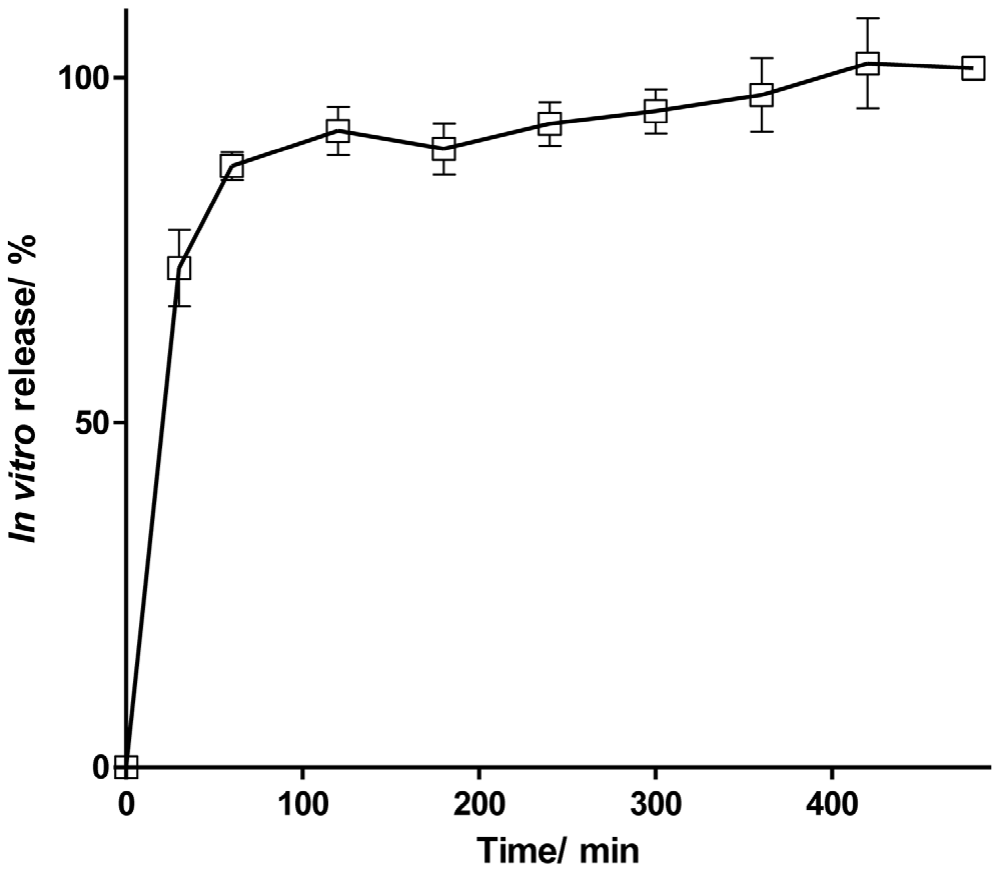
*In vitro* release profile of BIM from BI. Values expressed as mean ± SD.

The drug content in formulations was proved to be uniform. BIM was found at 9.009±0.030 µg/insert. CS inserts reached a controlled-release profile (greater than 4 hours). The **Fig. 5** showed the biphasic kinetic from release to BI. In the first stage it can be observed a burst release and subsequently an extended release of the drug. BI released 100% of the drug in 8 hours.

### Biodistribution studies

BIM was radiolabeled with radionuclide Tc-99m for biodistribution studies. The latter was chosen as it emits low-energy gamma rays that do not lead to serious health hazards. BIM was instantaneously labeled with ^99m^Tc. Radiolabeling procedure can yield two mainly radiochemical impurities, free technetium (^99m^TcO_4_^−^) and hydrolysed technetium (^99m^TcO_2_). In both mobile phases, ^99m^TcO_4_^−^reaches to the top of the ITLC strip (R_f_ = 0.9–1.0), whereas ^99m^TcO_2_ cannot travel much due to the difference in molecular weight and is retained at the base of the ITLC strip (R_f_ = 0.0). ^99m^Tc-BIM is a hydrophilic compound, which, similarly to ^99m^TcO_4_^−^,migrates to the top of the ITLC strip with saline and remains at the point of application when ethyl acetate/acetone (5/95) is used as eluent, like ^99m^TcO_2_. Then, saline was used to determine the amount of ^99m^TcO_2_, whereas ethyl acetate/acetone (5/95) was used to quantify ^99m^TcO_4_^−^. After preliminary BIM radiolabeling studies, which included the adjustment of labeling parameters, such as the amount of stannous chloride and pH, labeling procedure were optimized and an amount of 20 µg of stannous chloride and a pH of 7.0 were found to give the maximum labeling efficiency (89.38%), after purification.The results showed low levels of radiochemical impurities, allowing for images of better quality.

**Figure 6:**
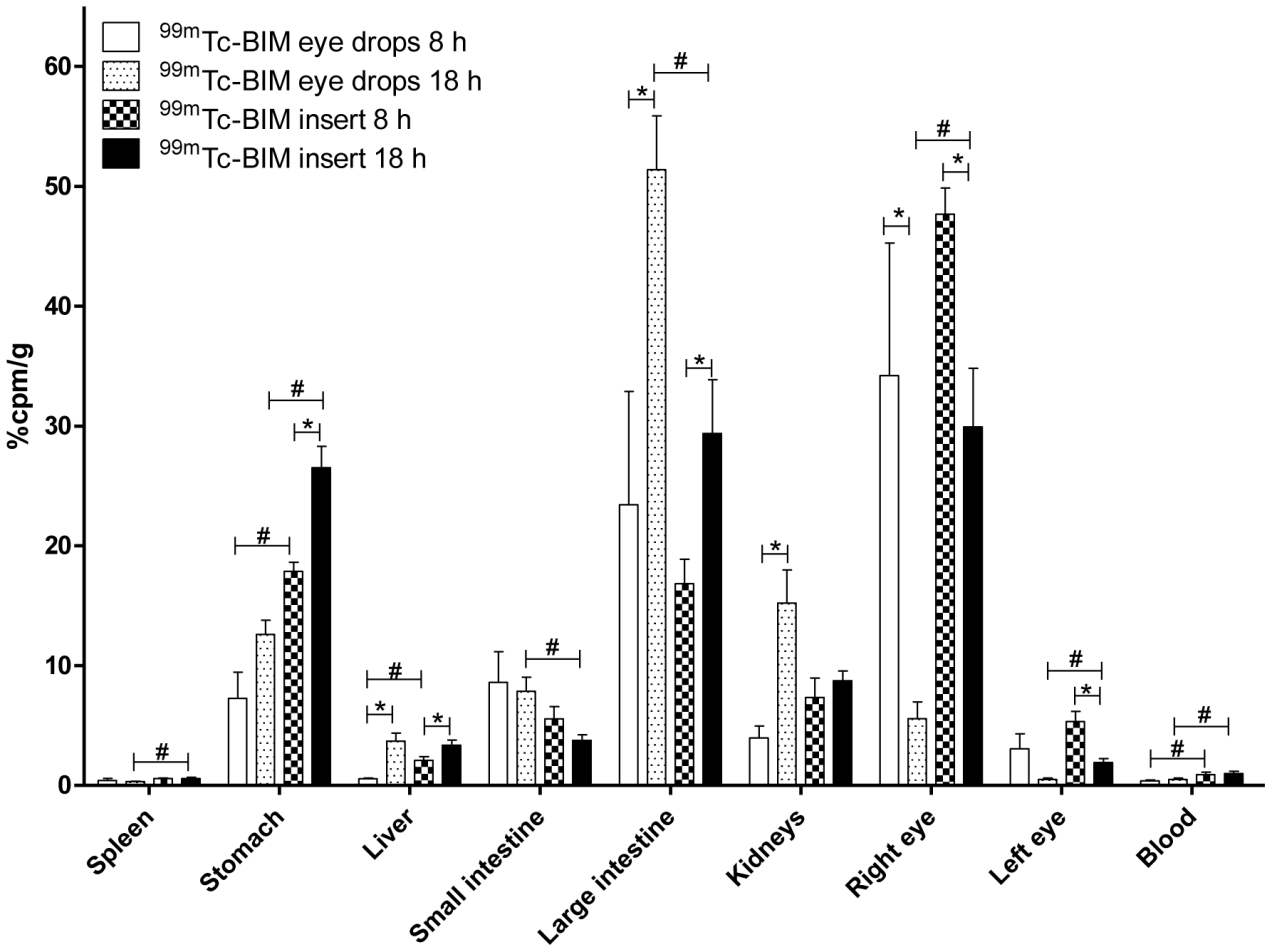
^99m^Tc-BIM biodistribution profile after eye drops instillation and chitosan inserts implantation. Values are expressed as ‘mean ± SD’ (*n* = 5). (*p<0.05 for 8 h vs. 18 h and ^#^p<0.05 for eye drops vs. inserts). Unpaired *Student t test*.

Results of biodistribution studies are presented in **Fig. 6**. At 8 h post-administration, 34.2±24.8% of ^99m^Tc-BIM from eye drops and 47.7±4.4% of ^99m^Tc-BIM from the inserts remained in the right eye. On the other hand, at 18 h, only 5.6±3.1% of ^99m^Tc-BIM from eye drops persisted in the right eye, whereas 29,9±10,9% of ^99m^Tc-BIM from the inserts was still present in the application site. In other words, the inserts prolonged retention of ^99m^Tc-BIM at the corneal site and reduced the extent of nasolacrimal drainage. The ^99m^Tc-BIM cleaned from the eye after eye drop instillation accumulated preferentially in the large intestine and in the kidneys, while ^99m^Tc-BIM cleaned from the eye after insert application accumulated preferentially in the stomach and in the large intestine.

### *In vivo* efficacy

**Figure 7:**
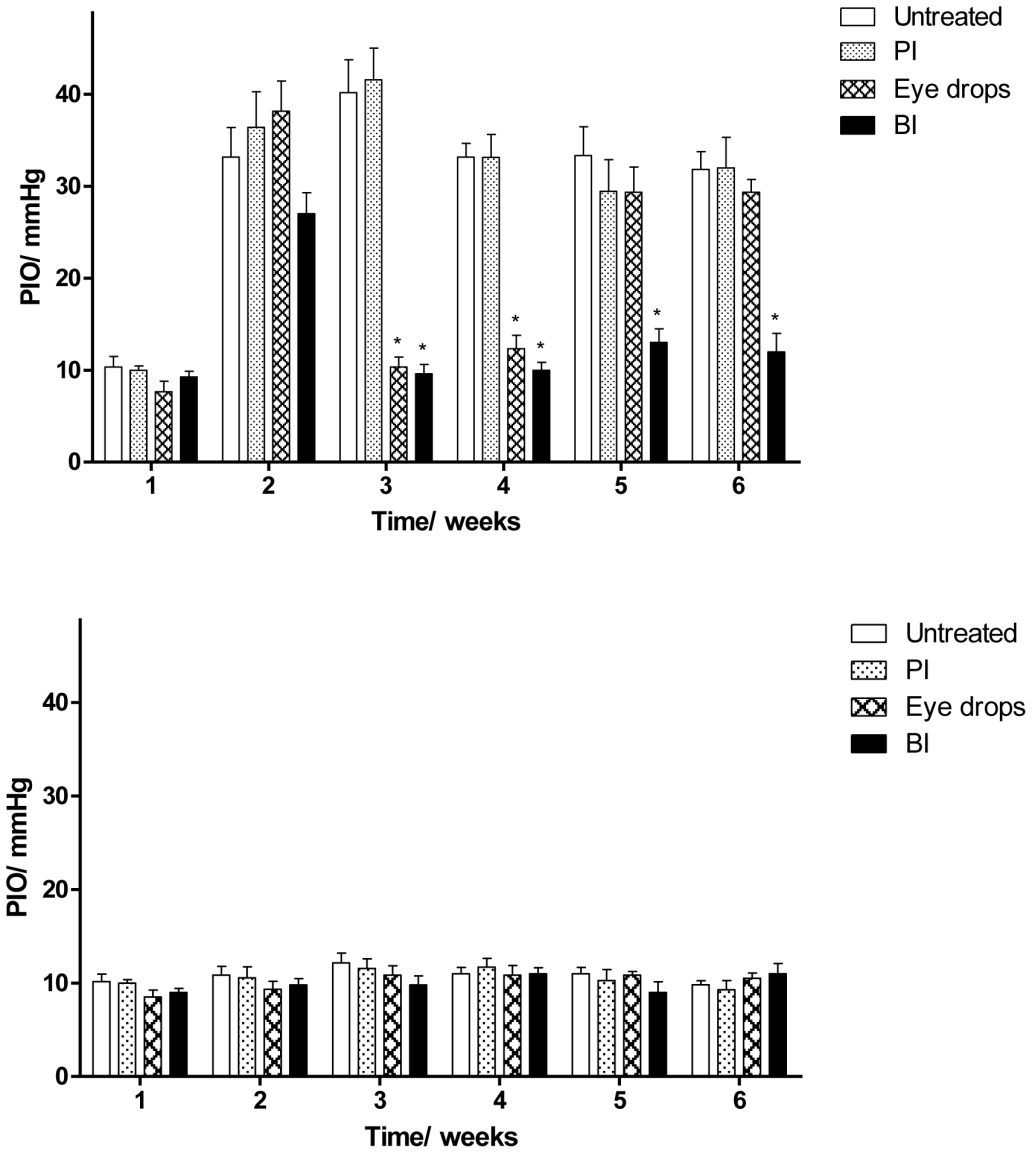
Effects of administration of BI on IOP. (a) Glaucomatous groups; (b) non-glaucomatous groups. Treatments initiated after confirmation of the elevated IOP, i.e. after second week. Values expressed as mean ± SD. *p<0.01 vs. untreated. One-way ANOVA followed by the Tukey post test.

**Figure 8:**
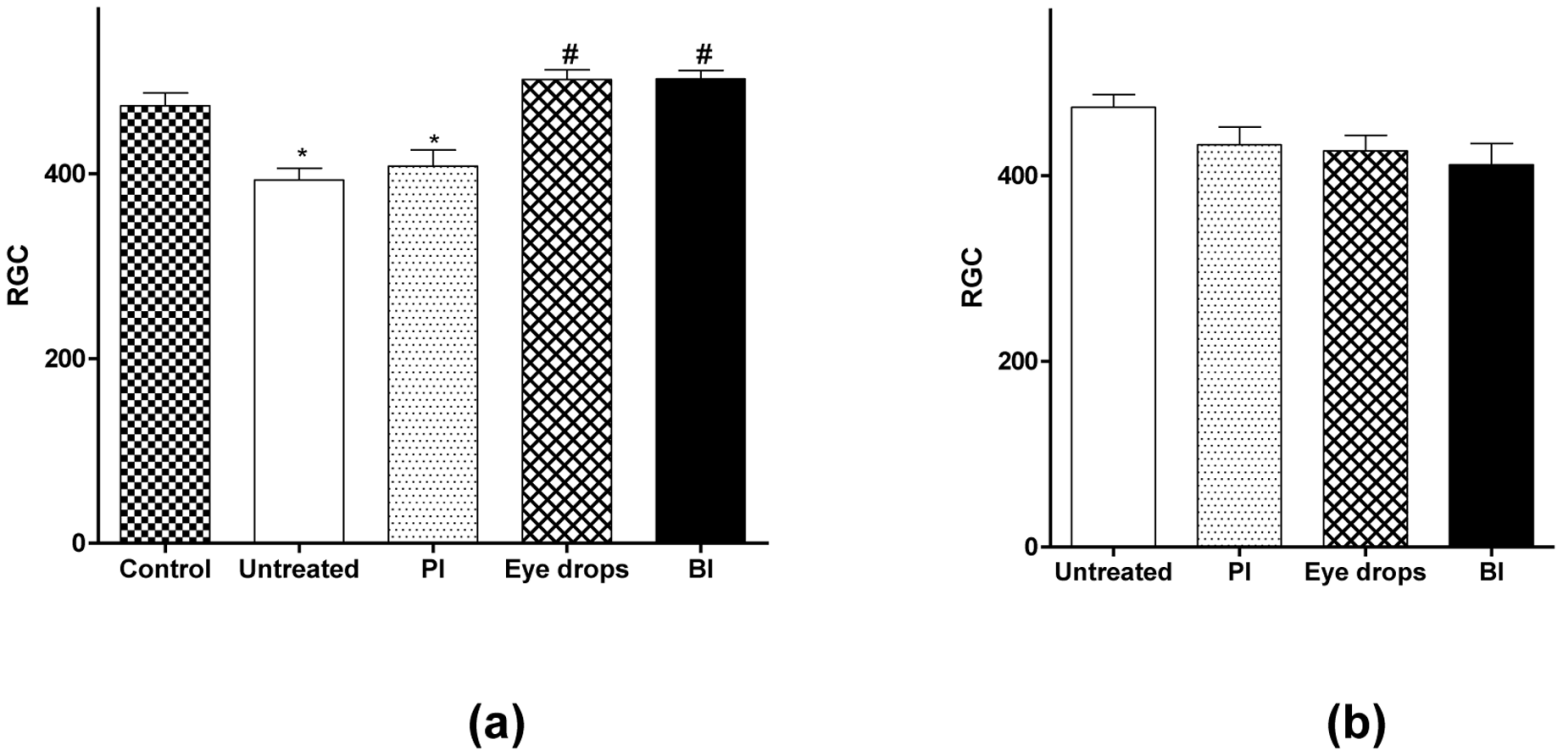
Effects of administration of BI on RGC counting. Quantification of RCG in retinas of (a) glaucomatous groups, compared to control (*p<0.05 vs. control and ^#^p<0.05 vs. untreated glaucoma) and (b) non-glaucomatous groups. Values expressed as mean ± SD. One-way ANOVA followed by the Tukey post test.

The ability of the inserts to controlled release BIM *in vivo* was tested in an experimental model of glaucoma induced by intraocular injection of HA. **Fig. 7** shows the IOP of all experimental groups during the period of 6 weeks (a, glaucomatous eye; b, normal eye). Before the first induction, the IOP of all the groups was at normal levels. After first induction, a significant increase in the IOP of all glaucomatous groups was observed. There was no difference between the groups (p = 0.2024). At this point, the treatment started. For the following four weeks after the treatment, it was observed that, while the IOP of non-treated glaucomatous animals and of glaucomatous animals treated with PI remained significantly high, the IOP of glaucomatous animals treated with BI was significantly lowered. The marketed formulation containing BIM reduced IOP for two weeks (period of eye drop instillation) but, when the treatment was interrupted, the IOP increased again. No significant changes in the IOP were induced by treatment of non-glaucomatous animals with BI or with marketed formulation (**Fig. 7** **b**). The anti-glaucomatous effects of the BI did not change the Mean Arterial Pressure (**Fig. 8**).

**Figure 9:**
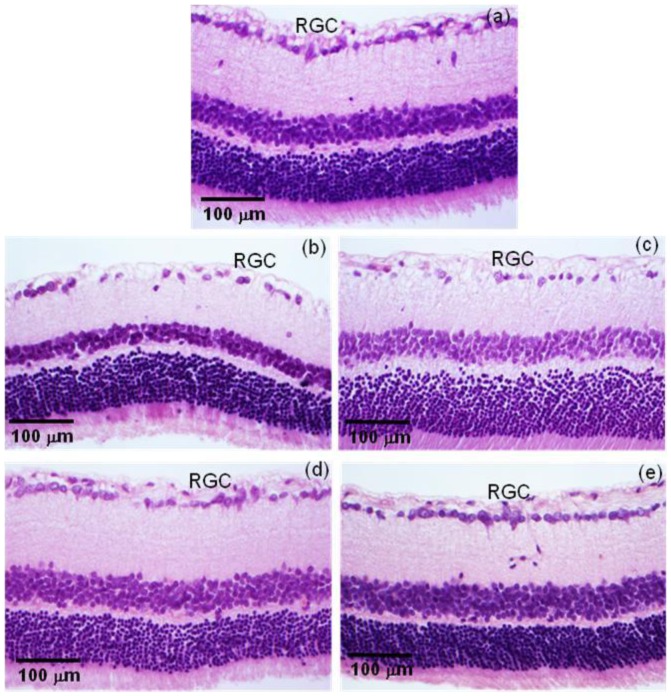
Histological analysis of retinal ganglion cells (RGC). Representative photomicrographs of retinas showing the smaller number of RCG in non-treated and PI-treated glaucomatous rats and the beneficial effect of BIM in this parameter. (a) Non-glaucomatous animals; (b) untreated glaucomatous animals; (c) PI glaucomatous animals; (d) BIM eye drops glaucomatous animals; (e) BI glaucomatous animals.

**Figure 10:**
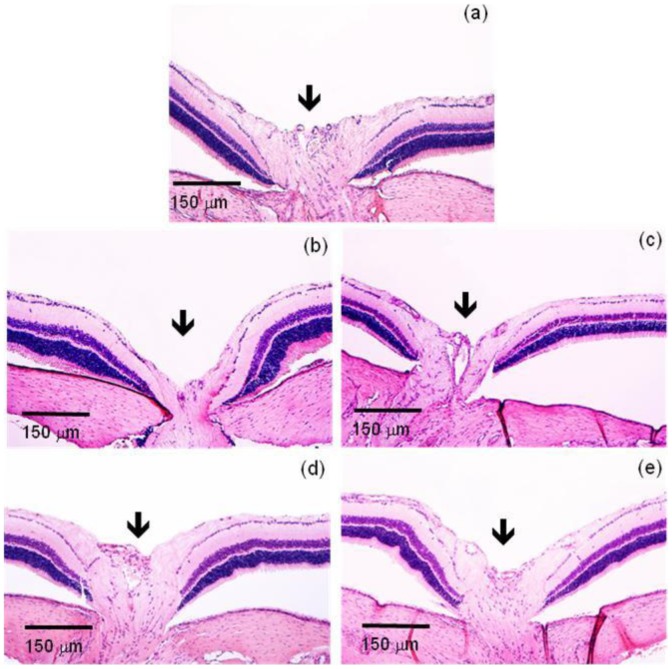
BIM induced neuroprotection in retinas of glaucomatous rats. Representative photomicrographs of excavation of the optic nerve (arrows). (a) Non-glaucomatous animals; (b) untreated glaucomatous animals; (c) PI glaucomatous animals; (d) BIM eye drops glaucomatous animals; (e) BI glaucomatous animals. Note an exacerbation of the excavation in untreated and PI glaucomatous animals (b and c) when compared with all other groups. This effect is reverted by BIM.

The IOP lowering effects of BI were followed by preservation of the RGC. As viewed in **Fig. 8** **a**, we found that non-treated glaucomatous animals and glaucomatous animals treated with PI showed a large reduction in the number of RGC (glaucomatous non-treated group: 393.2±31.5 cells; glaucomatous PI group 408.2±43.4 cells). Similar reduction was not noticed in glaucomatous animals treated either with marketed formulation containing BIM or with BI (473.5±27,9 cells in control group; 502.0±23,2 cells in the glaucomatous BIM eye drops group; 502.8±18,6 in glaucomatous BI group). Again, no significant changes were induced by BI in non-glaucomatous animals (**Fig 8** **b**). Representative histological images of retina showing RGC loss in glaucomatous groups are presented in **Fig. 9**. Additionally, the reduction in the number of RGC caused by elevated IOP led to a severe loss of neural fibers with consequent increase in the optic nerve head cupping (**Fig. 10**). These effects were abolished by the treatment with BI. Altogether, these data indicated that controlled release of BIM induced a neuroprotection by decreasing the loss of RGC and neural fibers.

During *in vivo* efficacy test, eventual eye irritation caused by the insert was evaluated. In all experiments, the inserts were well tolerated. No symptoms of ocular lesions, such as tearing, redness, edema, and inflammation, could be observed during the experimental assays. No anterior chamber inflammation or corneal changes were observed. Ocular surface structures and intraocular tissues proved to be normal. Furthermore, fluorescein staining did not indicate corneal or conjunctival ulcerations.

## Discussion

Ocular drug delivery has been a major challenge to pharmacologists and drug delivery scientists due to its unique anatomy and physiology [47]. An ideal therapy for chronic diseases, such as glaucoma, should maintain effective levels of drug for the longer duration following a single application [48]. Novel drug delivery strategies that provide controlled release for the treatment of such diseases and increase the patient's and doctor's convenience to reduce the dosing frequency and invasive treatment have been developed to sustain drug levels at the target site [47], [49]. Likewise, a great deal of attention is paid to develop non-invasive sustained drug release for eye disorders [48].

Within this context, we developed CS-based ocular inserts for sustained release of BIM, a highly efficacious ocular hypotensive agent [17]. CS, as well, seemed to be an adequate polymeric matrix due its biodegradable, nontoxic, biocompatible and mucoadhesive proprieties [29], [31]–[34]. Inserts were prepared as circular flexible membranes with 5 mm of diameter and about 50 µm of thickness (**Fig. 4**). Ocular insert used in animal or human clinical trial showed a thickness of 70 to 500 µm [50] suggesting that the inserts developed in our work have a suited breadth for clinical use.

Inserts hydrated very quickly (**Fig. 1**). BIM (hydrophilic drug) decrease the swelling index of BI, compared to PI. This fact, suggesting that there are intermolecular interactions between the drug and the polymeric matrix, is in agreement with the findings of Panomsuk *et al.* [51], in which the addition of mannitol to methylcellulose matrix also reduced the swelling index of the membranes. This result indicates the formation of hydrogen bonding between the drugs and the polymeric matrix. Therefore, the greater the number and strength of hydrogen bonding sites, the slower the diffusion of the water molecules in the hydrated matrix [51]. The presence of hydrogen bonding was further confirmed by ATR-FTIR spectra of BI (**Fig. 2**), in which O-H stretching bands were shifted to higher wave number. In this case, those changes provide evidence of the intermolecular interaction between the hydrophilic drug and polymeric matrix.

In DSC curves (**Fig. 3** **b**), it was observed that BIM addition dismembered the degradation peak of CS in two other peaks and increased the degradation temperature of the main chain of CS [45], [46]. This data indicates that the drug interacted with the matrix, in turn hampering the degradation of the matrix. Moreover, this finding corroborates those of ATR-FTIR spectra and swelling studies. These results suggest that the polymer and the drug interacted through hydrogen bonding.

Apart from the increase in O-H stretching wave number, no other changes in CS infrared spectrum were detected due the addition of BIM (**Fig. 2**), leading to the conclusion that there was no obvious chemical reaction between the drug and the matrix, in turn suggesting that the drug did not lose its activity in the drug-loaded inserts.

From morphological characterization (**Fig. 4**), it was not possible to identify crystallized particles in the middle or on the surface of the inserts containing BIM that could be attributed to drug crystals, suggesting that the drug was molecularly dispersed within the polymeric matrix. Both surface and lateral areas of inserts were homogeneous. The standard error of measurement (SEM) of drug content uniformity was very low (0,33%). It could be concluded that the drug dispersed uniformly throughout the inserts [52], as predicted by SEM analysis.

*In vitro* drug release studies (**Fig. 5**) indicated that there is a biphasic kinetic of BI release. This fact can be explained based on the effect of drug solubility and inserts hydratation. In this case, the drug used is highly soluble in aqueous and can pass through the porous structure of inserts. PBS was used as receptor fluid in the Franz cell and hydration of the inserts was very fast, as described above (80% in the first 20 min). These two factors together may have contributed to the burst effect observed.

On the other hand, *in vitro* drug release studies (**Fig. 5**) indicated that inserts were able to sustainedly release BIM. Although the drug is hydrophilic, the *in vitro* release data demonstrate that there is a certain time to release the drug content of the inner polymer matrix. As described above, this difficulty is associated with greater interpenetration of the polymer matrix in the main chain caused by greater interaction between the drug and the matrix itself, reducing the inflow of water into the matrix (after initial hydration) and consequently decreasing the release time of the drug.

Biodistribution studies were performed in order to evaluate the difference between ^99m^Tc-BIM and ^99m^Tc-BIM inserts drainage extension, at 8 and 18 hours post-ocular administration. Since the main route of drug elimination from the eye is nasolacrimal drainage, the gastrointestinal organs (stomach and intestines) were collected. Moreover, in order to evaluate possible absorption, blood and common organs of drug elimination (liver, spleen and kidneys) were also harvested. It was evidenced that CS was able to enhance precorneal retention time of BIM, which is probable due to the mucoadhesive proprieties of the polymeric matrix. It has already been proved that CS shows a prolonged precorneal residence time when delivered in the eye [53], [54]. Here, we demonstrated that CS was able to confer this property to BIM, most likely because of the physicochemical interaction between the drug and the polymeric matrix. An increment on the precorneal retention time of the drugs associated with polymeric matrix was reported by Gupta et al., when evaluated PLGA nanoparticles entrapping ^99m^Tc-sparfloxacin [55] or ^99m^Tc-levofloxacin [56] and PVA inserts entrapping ^99m^Tc-DTPA[57]. Preferable sites of ^99m^Tc-BIM accumulation were kidney and gastrointestinal tract (stomach, small intestine and large intestine), which are the two most important ways of elimination of BIM [58].

Reduction of IOP has long been the standard treatment for glaucoma [59]. Thereby, the effectiveness of the inserts was first evaluated by measuring the changes in IOP of glaucomatous rats caused by the inserts application. As expected, IOP lowering with conventional eye drop was only maintained during the daily treatment. As soon as the treatment was interrupted, IOP increased again. BI, on the other hand, were able to reduce IOP for four week after one application. These results showed a different drug delivery profile when comparing the in vitro and the in vivo experiments. It can be due to the fast in vitro drug release in the Franz cell system. So, the drug will be released constantly and fast, as described above. In the eye, such condition does not happen because the present liquid is the small and limited volume of the tears. Then, in this case, the drug will be released more slowly than in vitro In other words, our results suggest that therapeutic regimen of BIM could be reduced from daily to monthly.

If, on the one side, reduction of IOP is essential on treatment for glaucoma [59], by other, RGC damage is responsible for the loss of vision [14]. So, at the end of the treatment, we also evaluated histological sections of retina in optic nerve area in order to determine RGC loss and optic nerve head cupping. As predicted, both non-treated groups have showed significant RGC loss, while in both treated groups (eye drops or inserts), the RGC number was statistically equal to the number of non-glaucomatous animals, indicating that treatment with BIM also promoted neuroprotection [14]. It is important to underline that the same neuroprotection effect was obtained when BI was administered once or when BIM eye drops were administered daily for two weeks. The amount of BIM in one insert is equivalent to the amount of BIM in one drop.

In 2011, Robinson *et al.* developed polymeric systems for the sustained release of BIM [60], [61]. The main innovation of the present system, as compared with that developed by Robinson *et al.*, is that, while their system must be implanted in the anterior chamber of the eye, requiring a surgical procedure, the present system can simply be applied topically on the conjunctival sac, a non-invasive procedure. In 2013, Shafiee *et al*. proposed a DuraSite system to reduce the dosing frequency of BIM administration. BIM formulated in DuraSite system had ocular bioavailability superior to that of the conventional eye drops. Commercial DuraSite systems (AzaSite and Besivance) are still administered daily [62].

Natarajan *et al*., developed liposomes for sustained release of Latanoprost, a prostaglandin analog similar to BIM [61]. The sustained release of Latanoprost was achieved; however, the process for the production of liposomes still involves the use of organic solvents, which is undesirable in the pharmaceuticals industry. In 2013, Giarmoukakis *et al*. also developed biodegradable nanoparticles for sustained release of Latanoprot [23]. Unfortunately, invasive procedures were needed for periocular implantation of the developed formulation.

## Conclusions

The findings of this study revealed that CS-based ocular insert for the sustained release of BIM were successfully produced. A strong interaction between the drug and the polymer was achieved. The formulation enhanced precorneal residence time of the BIM, compared to conventional eye drops. The sustained release of BIM was proven by pharmacodinamic effects (IOP lowering and neuroprotection) and biodistribution studies. Consequently, after the data analysis, BI was able to sustain the release of BIM for over a month with only one dose applied. These results may reveal a potential application of this new formulation in glaucoma management, in order to improve patient compliance by lowering the frequency of administration and to enhance therapeutic effectiveness of glaucoma medical treatment.

## References

[1] Weinreb RN, Khaw PT (2004) Primary open-angle glaucoma. The Lancet 363: 1711–1720. 10.1016/S0140-6736(04)16257-0 15158634

[2] McKinnon SJ, Goldberg LD, Peeples P, Walt JG, Bramley TJ (2008) Current management of glaucoma and the need for complete therapy. Am J Manag Care 14: S20–27. 18284312

[3] Gupta SK, Niranjan G, Agrawal SS, Srivastava S, Saxena R (2008) Recent advances in pharmacotherapy of glaucoma. Indian Journal of Pharmacology 40: 197–208. 20040958 10.4103/0253-7613.44151 PMC2792620

[4] Salomao SR, Mitsuhiro MRKH, Belfort R Jr (2009) Visual impairment and blindness: an overview of prevalence and causes in Brazil. Anais Da Academia Brasileira De Ciencias 81: 539–549. 19722022 10.1590/s0001-37652009000300017

[5] Resnikoff S, Pascolini D, Etya'ale D, Kocur I, Pararajasegaram R, et al (2004) Global data on visual impairment in the year 2002. Bulletin of the World Health Organization 82: 844–851. 15640920 PMC2623053

[6] Boland MV, Quigley HA (2007) Risk factors and open-angle glaucoma: Classification and application. Journal of Glaucoma 16: 406–418. 17571004 10.1097/IJG.0b013e31806540a1

[7] Coleman AL, Miglior S (2008) Risk Factors for Glaucoma Onset and Progression. Survey of Ophthalmology 53: S3–S10. 19038621 10.1016/j.survophthal.2008.08.006

[8] Natu MV, Gaspar MN, Fontes Ribeiro CA, Cabrita AM, de Sousa HC, et al (2011) In vitro and in vivo evaluation of an intraocular implant for glaucoma treatment. International Journal of Pharmaceutics 415: 73–82. 21641984 10.1016/j.ijpharm.2011.05.047

[9] Bouhenni RA, Dunmire J, Sewell A, Edward DP (2012) Animal Models of Glaucoma. Journal of Biomedicine and Biotechnology. 10.1155/2012/692609 PMC3364028 22665989

[10] Casson RJ, Chidlow G, Wood JPM, Crowston JG, Goldberg I (2012) Definition of glaucoma: clinical and experimental concepts. Clinical and Experimental Ophthalmology 40: 341–349. 22356435 10.1111/j.1442-9071.2012.02773.x

[11] Cook C, Foster P (2012) Epidemiology of glaucoma: what's new? Canadian Journal of Ophthalmology-Journal Canadien D Ophtalmologie 47: 223–226. 22687296 10.1016/j.jcjo.2012.02.003

[12] Rafuse PE, Buys YM, Damji KE, Harasymowycz P, Lajoie C, et al (2009) Canadian Ophthalmological Society evidence-based clinical practice guidelines for the management of glaucoma in the adult eye Canadian Ophthalmological Society Glaucoma Clinical Practice Guideline Expert Committee. Canadian Journal of Ophthalmology-Journal Canadien D Ophtalmologie 44: S7–S54. 19492005 10.3129/cjo44s1

[13] Bean GW, Camras CB (2008) Commercially Available Prostaglandin Analogs for the Reduction of Intraocular Pressure: Similarities and Differences. Survey of Ophthalmology 53: S69–S84. 19038626 10.1016/j.survophthal.2008.08.012

[14] Takano N, Tsuruma K, Ohno Y, Shimazawa M, Hara H (2013) Bimatoprost protects retinal neuronal damage via Akt pathway. European Journal of Pharmacology 702: 56–61. 23395963 10.1016/j.ejphar.2013.01.038

[15] Brubaker RF (2001) Mechanism of action of bimatoprost (Luminan (TM)). Survey of Ophthalmology 45: S347–S351. 11434937 10.1016/s0039-6257(01)00213-2

[16] Burk RM, Woodward DF (2007) A historical perspective and recent advances in prostamide research and therapeutics. Current Opinion in Drug Discovery & Development 10: 413–421. 17659482

[17] Woodward DF, Krauss AHP, Chen J, Lai RK, Spada CS, et al (2001) The pharmacology of bimatoprost (Lumigan (TM)). Survey of Ophthalmology 45: S337–S345. 11434936 10.1016/s0039-6257(01)00224-7

[18] Liang Y, Woodward DF, Guzman VM, Li C, Scott DF, et al (2008) Identification and pharmacological characterization of the prostaglandin FP receptor and FP receptor variant complexes. British Journal of Pharmacology 154: 1079–1093. 18587449 10.1038/bjp.2008.142 PMC2440084

[19] Davies SS, Ju WK, Neufeld AH, Abran D, Chemtob S, et al (2003) Hydrolysis of bimatoprost (Lumigan) to its free acid by ocular tissue in vitro. Journal of Ocular Pharmacology and Therapeutics 19: 45–54. 12648303 10.1089/108076803762718105

[20] Gulsen D, Chauhan A (2005) Dispersion of microemulsion drops in HEMA hydrogel: a potential ophthalmic drug delivery vehicle. International Journal of Pharmaceutics 292: 95–117. 15725557 10.1016/j.ijpharm.2004.11.033

[21] Ludwig A (2005) The use of mucoadhesive polymers in ocular drug delivery. Advanced Drug Delivery Reviews 57: 1595–1639. 16198021 10.1016/j.addr.2005.07.005

[22] Ghate D, Edelhauser HF (2008) Barriers to glaucoma drug delivery. Journal of Glaucoma 17: 147–156. 18344762 10.1097/IJG.0b013e31814b990d

[23] Giarmoukakis A, Labiris G, Sideroudi H, Tsimali Z, Koutsospyrou N, et al (2013) Biodegradable nanoparticles for controlled subconjunctival delivery of latanoprost acid: In vitro and in vivo evaluation. Preliminary results. Experimental Eye Research 112: 29–36. 23603320 10.1016/j.exer.2013.04.007

[24] Aggarwal D, Kaur IP (2005) Improved pharmacodynamics of timolol maleate from a mucoadhesive niosomal ophthalmic drug delivery system. International Journal of Pharmaceutics 290: 155–159. 15664141 10.1016/j.ijpharm.2004.10.026

[25] Lee SS, Hughes P, Ross AD, Robinson MR (2010) Biodegradable Implants for Sustained Drug Release in the Eye. Pharmaceutical Research 27: 2043–2053. 20535532 10.1007/s11095-010-0159-x

[26] Aburahma MH, Mahmoud AA (2011) Biodegradable Ocular Inserts for Sustained Delivery of Brimonidine Tartarate: Preparation and In Vitro/In Vivo Evaluation. Aaps Pharmscitech 12: 1335–1347. 21979886 10.1208/s12249-011-9701-3 PMC3225539

[27] Kearns VR, Williams RL (2009) Drug delivery systems for the eye. Expert Review of Medical Devices 6: 277–290. 19419285 10.1586/erd.09.4

[28] Wadhwa S, Paliwal R, Paliwal SR, Vyas SP (2009) Chitosan and its Role in Ocular Therapeutics. Mini-Reviews in Medicinal Chemistry 9: 1639–1647. 20105127 10.2174/138955709791012292

[29] Zambito Y, Di Colo G (2010) Chitosan and its derivatives as intraocular penetration enhancers. Journal of Drug Delivery Science and Technology 20: 45–52.

[30] Chen RH, Lin JH, Yang MH (1994) RELATIONSHIPS BETWEEN THE CHAIN FLEXIBILITIES OF CHITOSAN MOLECULES AND THE PHYSICAL-PROPERTIES OF THEIR CASTED FILMS. Carbohydrate Polymers 24: 41–46.

[31] Felt O, Furrer P, Mayer JM, Plazonnet B, Buri P, et al (1999) Topical use of chitosan in ophthalmology: tolerance assessment and evaluation of precorneal retention. International Journal of Pharmaceutics 180: 185–193. 10370189 10.1016/s0378-5173(99)00003-4

[32] Gaserod O, Jolliffe IG, Hampson FC, Dettmar PW, Skjak-Braek G (1998) The enhancement of the bioadhesive properties of calcium alginate gel beads by coating with chitosan. International Journal of Pharmaceutics 175: 237–246.

[33] George M, Abraham TE (2006) Polyionic hydrocolloids for the intestinal delivery of protein drugs: Alginate and chitosan - a review. Journal of Controlled Release 114: 1–14. 16828914 10.1016/j.jconrel.2006.04.017

[34] Valenta C (2005) The use of mucoadhesive polymers in vaginal delivery. Advanced Drug Delivery Reviews 57: 1692–1712. 16182407 10.1016/j.addr.2005.07.004

[35] Rodrigues LB, Leite HF, Yoshida MI, Saliba JB, Cunha AS, et al (2009) In vitro release and characterization of chitosan films as dexamethasone carrier. International Journal of Pharmaceutics 368: 1–6. 18955123 10.1016/j.ijpharm.2008.09.047

[36] Perumal VA, Lutchman D, Mackraj I, Govender T (2008) Formulation of monolayered films with drug and polymers of opposing solubilities. International Journal of Pharmaceutics 358: 184–191. 18430529 10.1016/j.ijpharm.2008.03.005

[37] Eroglu H, Sargon MF, Oener L (2007) Chitosan formulations for steroid delivery: Effect of formulation variables on in vitro characteristics. Drug Development and Industrial Pharmacy 33: 265–271. 17454059 10.1080/03639040600713134

[38] Dobaria NB, Badhan AC, Mashru RC (2009) A Novel Itraconazole Bioadhesive Film for Vaginal Delivery: Design, Optimization, and Physicodynamic Characterization. Aaps Pharmscitech 10: 951–959. 19629707 10.1208/s12249-009-9288-0 PMC2802146

[39] (BRASIL) ANDVS, CRUZ FO (2010) Farmacopeia brasileira. Brasília: ANVISA: FIOCRUZ.

[40] Nunan EA, Cardoso VN, Moraes-Santos T (2002) Technetium-99m labeling of tityustoxin and venom from the scorpion Tityus serrulatus. Applied Radiation and Isotopes 57: 849–852. 12406627 10.1016/s0969-8043(02)00197-5

[41] de Barros ALB, Mota LdG, Ferreira CdA, Cardoso VN (2012) Kit formulation for 99mTc-labeling of HYNIC-βAla-Bombesin(7–14). Applied Radiation and Isotopes 70: 2440–2445. 22871450 10.1016/j.apradiso.2012.06.022

[42] Moreno MC, Marcos HJA, Croxatto JO, Sande PH, Campanelli J, et al (2005) A new experimental model of glaucoma in rats through intracameral injections of hyaluronic acid. Experimental Eye Research 81: 71–80. 15978257 10.1016/j.exer.2005.01.008

[43] Kumirska J, Czerwicka M, Kaczynski Z, Bychowska A, Brzozowski K, et al (2010) Application of Spectroscopic Methods for Structural Analysis of Chitin and Chitosan. Marine Drugs 8: 1567–1636. 20559489 10.3390/md8051567 PMC2885081

[44] Lawrie G, Keen I, Drew B, Chandler-Temple A, Rintoul L, et al (2007) Interactions between alginate and chitosan biopolymers characterized using FTIR and XPS. Biomacromolecules 8: 2533–2541. 17591747 10.1021/bm070014y

[45] Mucha M, Pawlak A (2005) Thermal analysis of chitosan and its blends. Thermochimica Acta 427: 69–76.

[46] Neto CGT, Giacometti JA, Job AE, Ferreira FC, Fonseca JLC, et al (2005) Thermal analysis of chitosan based networks. Carbohydrate Polymers 62: 97–103.

[47] Gaudana R, Ananthula HK, Parenky A, Mitra AK (2010) Ocular Drug Delivery. Aaps Journal 12: 348–360. 20437123 10.1208/s12248-010-9183-3 PMC2895432

[48] Gaudana R, Jwala J, Boddu SHS, Mitra AK (2009) Recent Perspectives in Ocular Drug Delivery. Pharmaceutical Research 26: 1197–1216. 18758924 10.1007/s11095-008-9694-0 PMC4516219

[49] Kuno N, Fujii S (2011) Recent Advances in Ocular Drug Delivery Systems. Polymers 3: 193–221.

[50] Lisa Land D, Benjamin WJ (1994) Sizes and shapes of conjunctival inserts. International Contact Lens Clinic 21: 212–217.

[51] Panomsuk SP, Hatanaka T, Aiba T, Katayama K, Koizumi T (1996) A study of the hydrophilic cellulose matrix: Effect of drugs on swelling properties. Chemical & Pharmaceutical Bulletin 44: 1039–1042.

[52] Semalty M, Semalty A, Kumar G (2008) Formulation and Characterization of Mucoadhesive Buccal Films of Glipizide. Indian Journal of Pharmaceutical Sciences 70: 43–48. 20390079 10.4103/0250-474X.40330 PMC2852059

[53] Yuan X-B, Li H, Yuan Y-B (2006) Preparation of cholesterol-modified chitosan self-aggregated nanoparticles for delivery of drugs to ocular surface. Carbohydrate Polymers 65.

[54] Kuntner C, Wanek T, Hoffer M, Dangl D, Hornof M, et al.. (2011) Radiosynthesis and Assessment of Ocular Pharmacokinetics of I-124-Labeled Chitosan in Rabbits Using Small-Animal PET. Molecular Imaging and Biology 13. 10.1007/s11307-010-0352-7 20526688

[55] Gupta H, Aqil M, Khar RK, Ali A, Bhatnagar A, et al.. (2010) Sparfloxacin-loaded PLGA nanoparticles for sustained ocular drug delivery. Nanomedicine-Nanotechnology Biology and Medicine 6. 10.1016/j.nano.2009.10.004 19857606

[56] Gupta H, Aqil M, Khar RK, Ali A, Bhatnagar A, et al (2011) Biodegradable levofloxacin nanoparticles for sustained ocular drug delivery. Journal of drug targeting 19: 409–417. 20678034 10.3109/1061186X.2010.504268

[57] Wilson CG, Zhu YP, Frier M, Rao LS, Gilchrist P, et al.. (1998) Ocular contact time of a carbomer gel (GelTears) in humans. British Journal of Ophthalmology 82. 10.1136/bjo.82.10.1131 PMC1722369 9924298

[58] Curran MP (2009) Bimatoprost A Review of its Use in Open-Angle Glaucoma and Ocular Hypertension. Drugs & Aging 26: 1049–1071. 19929032 10.2165/11203210-000000000-00000

[59] Lavik E, Kuehn MH, Kwon YH (2011) Novel drug delivery systems for glaucoma. Eye 25: 578–586. 21475311 10.1038/eye.2011.82 PMC3171267

[60] Robinson MR, Burke J, Schiffman R Treating ocular condition e.g. glaucoma, comprises providing biodegradable sustained release implants containing therapeutic agent e.g. bimatoprost and latanoprost and implanting the implants into the anterior chamber of an eye. Allergan Inc.

[61] Natarajan JV, Ang M, Darwitan A, Chattopadhyay S, Wong TT, et al (2012) Nanomedicine for glaucoma: liposomes provide sustained release of latanoprost in the eye. International Journal of Nanomedicine 7: 123. 22275828 10.2147/IJN.S25468 PMC3260956

[62] Shafiee A, Bowman LM, Hou E, Hosseini K (2013) Ocular pharmacokinetics of bimatoprost formulated in DuraSite compared to bimatoprost 0.03% ophthalmic solution in pigmented rabbit eyes. Clinical Ophthalmology 7: 1549–1556. 23940414 10.2147/OPTH.S48766 PMC3737010

